# Combined Intraperitoneal and Systemic Chemotherapy for Peritoneal Metastases: Drug Delivery Concepts, Pharmacokinetics, and Clinical Applications: A Narrative Review

**DOI:** 10.3390/pharmaceutics18020179

**Published:** 2026-01-30

**Authors:** Kohei Tamura, Joji Kitayama, Yasushi Saga, Yuji Takei, Hiroyuki Fujiwara, Hironori Yamaguchi, Ryozo Nagai, Kenichi Aizawa

**Affiliations:** 1 Department of Obstetrics and Gynecology, Jichi Medical University, 3311-1 Yakushiji, Shimotsuke 329-0498, Japan; 2 Department of Surgery, Jichi Medical University, 3311-1 Yakushiji, Shimotsuke 329-0498, Japan; kitayama.j@jihs.go.jp (J.K.);; 3 Department of Translational Research, Clinical Research Center, Jichi Medical University Hospital, 3311-1 Yakushiji, Shimotsuke 329-0498, Japan; 4 Jichi Medical University, 3311-1 Yakushiji, Shimotsuke 329-0498, Japan; 5 Clinical Pharmacology Center, Jichi Medical University Hospital, 3311-1 Yakushiji, Shimotsuke 329-0498, Japan

**Keywords:** intraperitoneal chemotherapy, peritoneal metastases, systemic chemotherapy, hyperthermic intraperitoneal chemotherapy, tumor microenvironment, clinical trials, tumor leukocyte ratio, cancer treatment, chemotherapy delivery, drug absorption

## Abstract

**Background/Objectives:** Peritoneal metastases (PMs) remain difficult to treat because the peritoneum–plasma barrier limits drug penetration from the systemic circulation. Intraperitoneal chemotherapy (IPC), particularly repeated intraperitoneal (IP) administration via implantable ports, can achieve high local drug exposure with prolonged retention. This review summarizes the pharmacological rationale, clinical evidence, and future directions of catheter-based IPC, with emphasis on combined IP and systemic chemotherapy for ovarian, gastric, and pancreatic cancers. **Methods:** We narratively reviewed prospective clinical trials and key retrospective studies evaluating IPC and compared repeated catheter-based IPC with hyperthermic intraperitoneal chemotherapy (HIPEC) and pressurized intraperitoneal aerosol chemotherapy (PIPAC). Efficacy, safety, practice considerations, and opportunities for ascites-based monitoring were examined. **Results:** In ovarian cancer, several randomized trials demonstrated improved progression-free survival (PFS), and in selected trials, improved overall survival (OS) was demonstrated using IP plus intravenous (IV) therapy, although in the latter trials, toxicity and catheter-related complications limited treatment completion. A phase III Intraperitoneal Therapy for Ovarian Cancer with Carboplatin (iPocc) trial further showed significantly prolonged PFS with IP carboplatin and weekly paclitaxel, with non-catheter-related toxicity comparable to that of IV therapy. In gastric and pancreatic cancer, phase II studies reported symptomatic control, cytologic conversion, and higher rates of conversion surgery in selected patients, although confirmatory phase III data are limited. Device complications, including infection, obstruction, and leakage, occurred, but were manageable. **Conclusions:** Repeated catheter-based IPC is a feasible approach that enhances intraperitoneal drug delivery and complements IV chemotherapy. Future priorities include randomized trials, pharmacokinetic optimization, and biomarker-guided patient selection, supported by serial ascites assessment to refine indications and improve outcomes.

## 1. Introduction

Recent clinical trials have explored intraperitoneal chemotherapy (IPC) as a potential treatment to improve prognoses for patients with peritoneal metastases (PMs) [1,2,3,4,5,6]. While some studies have demonstrated promising results, outcomes remain unsatisfactory in many cases [7,8,9]. Several factors contribute to treatment success, including the choice of anti-cancer drugs, drug concentrations in both serum and tumor tissues, specific administration routes, and management of adverse effects [10,11,12,13]. These factors are critical for enhancing treatment efficacy against PMs.

IPC is considered a rational local-regional therapy for PMs, offering distinct advantages such as higher drug concentrations in the peritoneal cavity compared to IV administration. This direct approach targets PMs and free intraperitoneal cancer cells, leading to increased drug infiltration and prolonged drug half-lives [14,15,16,17]. In contrast, systemic chemotherapy via IV administration often suffers limitations in treating PMs. The peritoneum–plasma barrier restricts effective delivery of drugs from blood vessels to the peritoneum, resulting in insufficient drug concentrations at metastatic sites [11].

However, systemic chemotherapy has its own advantages. It achieves higher peak concentrations of anti-cancer drugs in the serum, distributes drugs throughout the entire body to suppress distant metastatic disease, and ensures higher drug delivery to primary tumors with adequate blood flow, such as those in the liver, spleen, ovary, and digestive organs [11,17,18], as well as to tumor cores. Given these considerations, combining IP and systemic chemotherapy emerges as a promising approach for treating PMs of various types.

At present, three principal forms of IPC are used in clinical practice: hyperthermic intraperitoneal chemotherapy (HIPEC), pressurized intraperitoneal aerosol chemotherapy (PIPAC), and catheter-based repeated IP drug administration. As numerous comprehensive reviews have already addressed HIPEC and PIPAC, the present review focuses mainly on catheter-based IPC, providing an integrated overview of its biological rationale, therapeutic potential, and current clinical challenges in management of PMs. In addition, we discuss future perspectives for optimizing IPC as a core component of multimodal treatment strategies for PMs.

## 2. Methods

We conducted a comprehensive literature search as part of this narrative review. The primary database queried was PubMed, supplemented with searches in the Cochrane Library for relevant trials. Key search terms included combinations of “IPC”, “systemic chemotherapy”, “PMs”, “PTX” and related terms, e.g., “gastric cancer” for disease context. The search was limited to studies published from approximately January 2010 to 2025, ensuring a focus on contemporary evidence. We imposed no language restrictions on the initial search, but only full-text articles available in English were reviewed. Relevant articles cited by retrieved papers were also screened to avoid missing any major studies.

We targeted clinical studies at evidence levels of Phase II or higher, as well as pertinent meta-analyses that evaluated IPC with or without systemic therapy for PMs. Studies reporting pharmacokinetics, treatment efficacy on prognosis, response rates, or safety/toxicity profiles were prioritized. In particular, we included Phase II single-arm trials, Phase III randomized controlled trials (RCTs) and large cohort studies that provided quantitative outcome data. Exclusion criteria encompassed case reports and small case series with very limited patient numbers, as such reports provide insufficient evidence for a clinical review. When multiple reports stemmed from the same patient cohort, the most comprehensive or recent publication was evaluated.

Consistent with a narrative review approach, no formal risk of bias assessment was undertaken for these studies. However, study design and quality were considered qualitatively during analysis. For instance, evidence from RCTs was weighed more heavily than that from single-arm or cohort studies. This approach allowed us to synthesize literature while being mindful of potential biases, even though an explicit standardized bias scoring was not performed. We acknowledge that the narrative methodology carries a risk of selection bias, but we have striven to mitigate this with broad database coverage and careful, critical appraisal of included studies.

## 3. Benefits of the Intraperitoneal Approach

IP administration of anticancer drugs offers several advantages due to the anatomical characteristics of the peritoneum, which is an enclosed space. Specifically, (a) IP chemotherapy directly targets both free cancer cells in the peritoneal cavity and PMs. (b) IPC achieves higher drug concentrations in the peritoneal cavity than IV administration, as the peritoneum–plasma barrier prevents intravenously administered drugs from entering the abdominal cavity [19]. (c) Most drugs administered via IPC are not readily absorbed into the systemic circulation, leading to prolonged drug half-lives in the peritoneal cavity, which may reduce systemic toxicity, such as anemia and febrile neutropenia [20]. (d) IPC enables deeper infiltration of tumors. Furthermore, (e) IPC can be repeatedly administered using an access port and an intraperitoneal catheter, allowing for outpatient treatment.

HIPEC involves administering heated chemotherapy fluid, often following cytoreductive surgery (CRS), and is perfused using specialized equipment [19]. HIPEC has the benefit that it enhances chemotherapeutic efficacy by improving drug penetration and tumor cytotoxicity, achieving high local drug concentrations [21]. HIPEC + CRS has shown notable efficacy in treating PMs from various malignancies, including gastric cancer (GC) [22,23], ovarian cancer [6,24], colorectal cancer [25,26], and pseudomyxoma peritonei [27,28]. In Western countries, HIPEC is a standard treatment for PMs, whereas in other countries, it is not yet considered a replacement for systemic chemotherapy. Several factors contribute to this, including the one-time nature of the treatment combined with CRS, the lack of large prospective studies, the high frequency of severe side effects, the need for specialized equipment and techniques, the high cost and ambiguous indications for patient selection. For instance, in East Asia, gastric cancer is common [29], and clinical trials of HIPEC have been evaluated as a treatment option [30,31]. However, the median overall survival (OS) in these trials was less than one year, and the one-year survival rate did not exceed 50%, which is no better than the results of systemic chemotherapy.

PIPAC involves pressurized delivery of aerosolized anticancer drugs, such as doxorubicin (Doxo) or platinum agents like cisplatin (CDDP) or oxaliplatin (L-OHP), into the peritoneal cavity at a pressure of 12 mmHg for 30 min. This newly introduced method enables uniform distribution of the drug throughout the peritoneum and PMs [32]. Although PIPAC has shown some efficacy against various malignancies, it has not yet been integrated as a standard therapy [33,34]. All clinical trials of PIPAC have been Phase I or II studies. Phase I studies focused on safety and pharmacokinetics of drugs such as L-OHP, CDDP, Doxo, and nab-paclitaxel [35,36,37], whereas Phase II studies demonstrated safety and partial efficacy of PIPAC against PMs from gastric, colorectal, and ovarian cancers. Moreover, some observational studies have indicated that PIPAC is safe and feasible for various tumors [38,39,40,41]. However, these studies have been limited in size and scope, making it difficult to assess whether PIPAC is efficacious for PMs. Further basic and Phase I/II studies are necessary to ensure safety and to identify effective drugs for PMs; however, ongoing Phase III trials are investigating PIPAC’s role in gastrointestinal and ovarian carcinoma [42,43].

## 4. Disadvantages of the Intraperitoneal Approach

There are several disadvantages associated with IPC. In particular, HIPEC has significant drawbacks in terms of toxicity and procedural complexity. Perioperative morbidity remains a major concern. Comprehensive reviews report that as many as one-third of patients experience serious complications following CRS + HIPEC. Common adverse events include severe hematologic toxicities such as neutropenia and gastrointestinal complications such as anastomotic leaks, postoperative fistulas and prolonged ileus. These risks and the need for specialized surgical expertise and intensive perioperative care have prevented adoption of HIPEC [44]. Accordingly, rigorous multidisciplinary evaluation and patient counseling are imperative to ensure that the anticipated survival benefit of HIPEC surpasses the potential for serious complications in individual cases.

In catheter-based IPC, relatively small volumes of anticancer agents are repeatedly administered via a subcutaneously implanted access port in the lower abdomen, which is connected to an intraperitoneal catheter. Drugs are dissolved in approximately 1000 mL of saline at normothermic temperature and infused until completion of the treatment. Consequently, systemic toxicity is markedly lower than with HIPEC. However, the device needs to be implanted laparoscopically or under laparotomy in the operating room, with the tip of the catheter placed in the pouch of Douglas. The procedure is typically completed within 1 h with minimal bleeding. However, it requires a certain level of expertise and carries potential complications associated with the port system, such as infection, catheter obstruction, and subcutaneous fluid leakage.

Phase II/III studies involving more than 40 patients conducted since 2020 have reported complication frequencies. We selected studies to investigate complications, as past studies may differ in port establishment techniques and antibiotic choice, which could influence the incidence of complications such as bleeding and infection. Small studies are not reliable. In retrospective studies in Phase II/III for gastric cancer, no complications were reported [45,46], while three Phase III studies showed port complication rates of 23%, 13.6%, and 30.4% [4,47,48]. Specifically, Chia et al. reported that the number of Common Terminology Criteria for Adverse Events (CTCAE) grade ≤ 2 and grade 3 port leakage or infection were *n* = 6 and 4, respectively, with grade 3 complications requiring port re-siting or removal [47]. Our institution reported grade 3 complications, including port obstruction and infection, with n = 3 for both [48]. Yamada et al. reported no CTCAE grade ≤ 2 complications, and one grade 3 complication, though details of grade 3 were not specified [4]. While these statistics are rough, the complication rate across all grades was 11.4% (n = 30), with greater than grade 3 complications at 4.2% (n = 11): obstruction 1.1%, infection 3.0%, and leakage 1.9%. Furthermore, IPC has additional disadvantages. Repeated administration or postoperative peritoneal alterations may induce adhesions, causing heterogeneous IP drug distribution, thereby attenuating antitumor efficacy [15]. In addition, malignant ascites may negatively influence treatment outcomes, as fluctuations in ascitic volume and composition can influence drug concentration and residence time in the peritoneal cavity, ultimately leading to reduced therapeutic benefit [16,49]. Although IP chemotherapy can result in complications, the incidence is relatively low and manageable. Advantages of IPC far outweigh its disadvantages. The convenience of the port device facilitates repeated IP administration, reducing preparation time for each chemotherapy session. Moreover, the device provides a way to evaluate the treatment effect of IPC, as ascitic samples can easily and non-invasively be obtained through the port to examine the presence of tumor cells and tumor/leukocyte ratio in ascites during treatment. This is useful for understanding the tumor microenvironment (TME) and for estimating therapeutic effects and prognosis. It may also help to determine timing of conversion surgery for various tumor types [50,51]. To facilitate understanding of the pharmacological and clinical characteristics of IPC, the major advantages and disadvantages of this approach are summarized in Table 1.

## 5. Combination with Systemic Chemotherapy

Systemic chemotherapy offers several advantages, including the ability to deliver anticancer agents to extraperitoneal lesions and to eradicate microscopic systemic disease, thereby contributing to overall disease control and prevention of recurrence. Numerous clinical trials have been conducted, resulting in establishment of a wide range of therapeutic regimens [44]. Because there are no adhesions or localized barriers in the vascular system, pharmacokinetics of intravenously administered drugs are generally considered highly reproducible. While IPC primarily targets tumor surfaces, systemic administration allows anticancer agents to reach tumor cores via the bloodstream [8]. Furthermore, systemic chemotherapy typically achieves higher peak plasma concentrations of cytotoxic agents than with IPC, which may enhance therapeutic efficacy at distant metastatic sites [18]. However, in addition to the previously described peritoneal barrier, systemic chemotherapy has several inherent limitations. Its plasma half-life is generally shorter than that observed with IPC [52]. Systemic toxicities occur more frequently, often necessitating dose reductions or treatment interruptions that compromise the intended dose intensity. Moreover, systemic chemotherapy tends to have a slower onset in reducing malignant ascites, sometimes requiring prolonged treatment before achieving symptomatic improvement in patients with peritoneal PMs [53].

**Figure 1:**
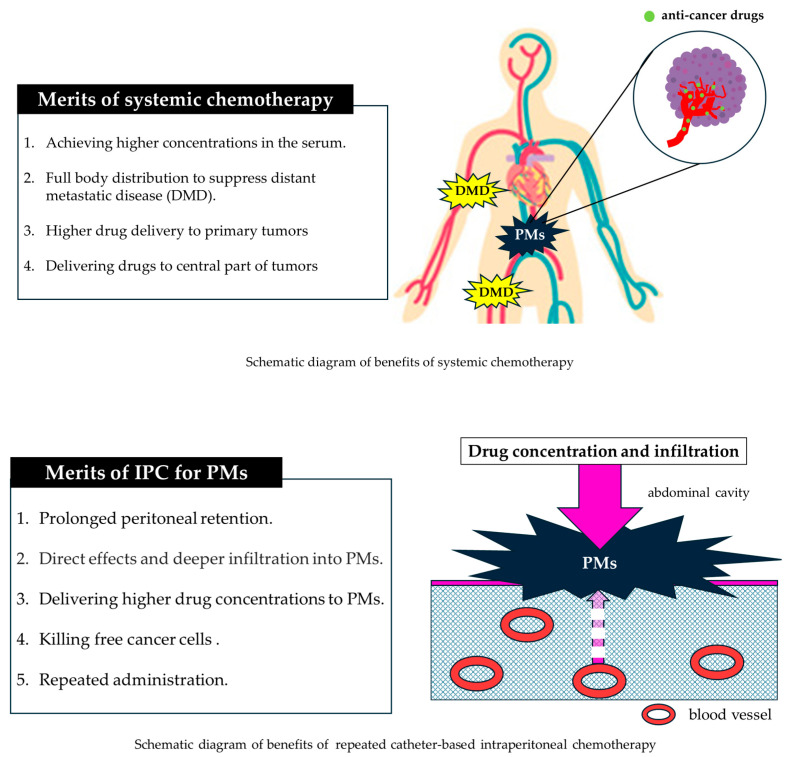
Advantages of systemic and intraperitoneal chemotherapy for PMs.

Taken together, both IPC and systemic chemotherapy possess distinct advantages in managing PMs (Figure 1). Accordingly, combined therapeutic strategies that integrate both modalities are promising, and numerous clinical studies have been undertaken to evaluate their synergistic efficacy.

## 6. Clinical Trials of Intraperitoneal Chemotherapy by Cancer Type

### 6.1. Ovarian Cancer

Ovarian cancer is the second most common gynecological malignancy and is a leading cause of death. Approximately 314,000 women are newly diagnosed each year. Ovarian cancer often spreads rapidly into the peritoneal cavity, leading to PMs, as the ovaries are largely exposed to the peritoneal cavity. Routes and ease of access to PMs in ovarian cancer differ from those of most gastrointestinal cancers, as the latter typically originate in the lumen and require serosal rupture to form PMs. Thus, around 80% of patients with ovarian cancer are diagnosed in advanced stages (Stage III and IV) due to the lack of symptoms in early stages. As a result, 207,000 patients die annually worldwide, with five-year survival rates of 27% and 13%, respectively [54].

Standard therapeutic strategies are primary debulking surgery (PDS) and adjuvant systemic chemotherapy with a combination of platinum and taxane for advanced ovarian cancer to prevent recurrence or to shrink residual tumors. Most patients achieve remission initially; however, this status is temporary, and more than half of patients experience relapse in the abdominal cavity with low chemosensitivity, ultimately leading to death [55,56]. Therefore, identifying the optimal route of administration and the most effective anti-cancer drugs to suppress disease progression is crucial.

Four randomized trials investigated whether IPC is superior to IV administration for advanced ovarian cancer. Alberts et al. showed that IP CDDP is superior to IV CDDP in terms of OS. In that study, 546 patients with FIGO Stage III who underwent surgeries involving laparotomy with at least bilateral salpingo-oophorectomy, hysterectomy, omentectomy, and resection of tumor nodules and residual tumors measuring 2 cm or less were assigned to two groups. Regimens in both groups involved administration of IV cyclophosphamide (CPA) 600 mg/m^2^ of body surface area plus either IP CDDP 100 mg/m^2^ or IV CDDP 100 mg per square meter. OS in the IP group was significantly longer than that in the IV group (49 months vs. 41 months, *p* = 0.02) [57].

A second Phase III study compared benefits of IV CDDP and paclitaxel (PTX) with IV carboplatin (CBDCA) and PTX plus IP CDDP in 462 patients with residual tumors measuring less than 1.0 cm. The control group received IV PTX 135 mg/m^2^ followed by IV CDDP 75 mg/m^2^ every three weeks for six courses. The experimental group received IV CBDCA area under the curve (AUC) 9 for two courses, plus IV PTX 135 mg/m^2^ followed by IP CDDP 100 mg/m^2^ every three weeks for six courses. Progression free survival (PFS) in the experimental group was superior to that in the control group (28 months vs. 22 months, *p* = 0.01), and OS showed a near-significant improvement (63 months vs. 52 months, *p* = 0.05). The study suggested that IPC holds promise as a treatment option [58].

The third randomized trial, by Armstrong et al., compared efficacy of IPC with IV PTX and CDDP against IV PTX plus IP PTX and CDDP. Patients with Stage III ovarian carcinoma or primary peritoneal carcinoma with residual tumors smaller than 1.0 cm were eligible. The IV group received IV PTX 135/m^2^ followed by IV CDDP 75 mg/m^2^, whereas the IP group received IV PTX with the same dose, plus IP CDDP 100 mg and PTX 60 mg/m^2^. Treatment was administered every three weeks for six cycles [59]. Results of this study were outstanding. PFS in the IV and IP groups was 18.3 months and 23.8 months, respectively (*p* = 0.05). OS was 49.7 months in the IV group and 65.6 months in the IP group (*p* = 0.03). However, fewer than half of IP group patients completed six cycles due to the treatment burden. The incidence of CTCAE Grade 3 and 4 side effects, such as fatigue, pain, and hematologic and neurotoxicity, was higher in the IP group than in the IV group. Additionally, the quality of life was clearly worse in the IP group before cycle 4 and three to six weeks after treatment.

Three randomized trials suggest that IPC has potential to improve prognosis for ovarian cancer with PMs. However, there are limitations when comparing regimens of IV therapy and IP therapy. The first study did not include PTX, a key drug in ovarian cancer, and regimens in the other trials did not precisely compare effects of IP and IV treatments, as the IP group received more intensive chemotherapy, and drug doses were not the same between the two groups, complicating interpretation of results.

Recently, Intraperitoneal Therapy for Ovarian Cancer with Carboplatin (iPocc) trials from Japan have shown the superiority of IP in PFS when comparing a simple control and an experimental group. The trial involved 655 newly diagnosed ovarian cancer patients with FIGO Stage II to IV who underwent laparotomy or laparoscopy surgery and were randomized into two groups, regardless of residual tumor size. All patients received IV PTX 80 mg/m^2^ on days 1, 8, and 15 of a 21-day cycle. In addition, patients in the control group received IV PTX plus IV CBDCA AUC 6 every three weeks, whereas patients in the experimental group received IV PTX plus IP CBDCA AUC 6 every three weeks for six to eight cycles. PFS in the IP group was longer than in the IV group (23.5 months vs. 20.7 months, *p* = 0.04), though OS was not statistically different (64.9 months vs. 67.0 months). Another key finding was that the incidence of non-catheter-related adverse events did not differ statistically in the IP and IV groups, despite the occurrence of catheter-related complications such as catheter infections [2]. Notably, in most previous studies, IP regimens used CDDP, and the iPocc trial was the first large study to use IP CBDCA. Improvement in prognosis was observed regardless of residual tumor diameter. This trial indicates that IPC may be a promising treatment for advanced ovarian cancer to improve prognosis. Future randomized trials should refine OS by determining the best timing, dose, and type of anti-cancer drugs for IPC.

### 6.2. Pancreatic Cancer

Pancreatic cancer is one of the leading causes of cancer-related death, with approximately 466,000 estimated deaths worldwide, showing considerable variation [60]. The five-year survival rate is less than 10% for all pancreatic cancer cases, as 80% of patients are diagnosed with PMs or metastases that exclude them from resection [61,62]. Even for patients undergoing curative R0 resection, the five-year survival rate is only about 20% [63]. Among 2924 patients, the incidence of peritoneal metastases (PMs) was approximately 9%, ranking second only to liver metastases [64]. Mackay et al. researched the correlation between the primary origin and metastasis sites among 9952 patients with metastatic pancreatic cancer in the Netherlands between 2005 and 2015. PMs alone were observed in 7.7% of cases. One-year OS for patients with PMs from the head, body, and tail of the pancreas was 13.9%, 11.8%, and 10.5%, respectively, with median survival being 3.4, 2.3, and 2.2 months (*p* = n.s) [65]. Takahara et al. found that 15% of 494 advanced pancreatic cancer patients had malignant ascites (MA), with a median OS of only 6 weeks. The study concluded that the impact of MA on prognosis is comparable to that of microscopic PMs [66]. Ferrone et al. reported OS of 7 months in patients with PMs and 6 months in those with locally advanced cancer who had positive peritoneal washing cytology [67]. These findings underscore the poor prognosis of pancreatic cancer with PMs, highlighting the need for promising therapies.

Currently, standard systemic chemotherapy regimens for metastatic pancreatic cancer are fluorouracil, leucovorin, irinotecan (CPT-11), and L-OHP (FOLFORINOX) or gemcitabine (GEM) plus nab-paclitaxel (nab-PTX). These regimens are compared to GEM monotherapy, which was the first-line therapy before the 2010s. OS in the trial and control groups was about 8–11 and 6 months, respectively (*p* < 0.001). For PFS, these periods were 5–6 and 3 months (*p* < 0.001). Although OS and PFS were significantly longer in the trial group, the incidence of grade 3/4 adverse events such as neutropenia, febrile neutropenia (FN), thrombocytopenia, diarrhea, fatigue, and neuropathy was notably higher [68,69]. However, there is no definitive evidence regarding efficacy of FOLFIRINOX or GEM plus nab-PTX for improving the prognosis of patients with PMs. In fact, the majority of patients with PMs eventually develop malignant ascites, which often leads to complications such as bowel obstruction and hypoalbuminemia. These conditions frequently preclude continuation or even initiation of systemic chemotherapy. Therefore, new regimens to manage PMs and ascites are essential.

HIPEC for PMs in patients with PDAC has been poorly studied given the aggressive nature of pancreatic cancer and its overall poor prognosis. However, two studies have reported favorable outcomes in patients who received catheter-based IPC using PTX. In a Phase II study, Satoi et al. showed that the combination of IP and IV chemotherapy holds promise for pancreatic cancer with PMs. The regimen in that study consisted of IV and IP PTX 50 mg and 20 mg/m^2^ on days 1 and 8. S-1, an oral chemotherapy consisting of tegafur, gimeracil and oteracil, was administered at 80 mg/m^2^ for 14 consecutive days, followed by 7 days of rest. OS was 16.3 months, with 1-year and 2-year survival rates of 62% and 23%, respectively. The response rate and disease control rate were 36% and 82%. When compared to systemic chemotherapy studies for pancreatic cancer with PMs, these results suggest that the combination of IP and IV therapy may be more effective than systemic chemotherapy alone. Notably, OS was significantly longer in patients who underwent conversion surgery compared to those who did not (27.8 vs. 14.2 months, *p* = 0.0038). This finding suggests that appropriate patient selection for conversion surgery is crucial for improving prognosis and that in carefully selected cases, this procedure may achieve outcomes comparable to those of upfront resection in patients with resectable pancreatic cancer. As for adverse events, grade 3/4 hematologic toxicities such as neutropenia, leukopenia, and FN occurred in 42% of patients, while non-hematologic adverse events like appetite loss, nausea, vomiting, diarrhea, and mucositis were also observed [70].

In a second study, Yamada et al. investigated a new regimen combining IP and IV chemotherapy in 46 patients with PMs or positive peritoneal cytology detected during staging laparoscopy or laparotomy in a Phase II study. The recommended regimen consisted of IV GEM (800 mg) and nab-PTX (75 mg), and IP PTX (20 mg/m^2^), respectively. IV GEM and nab-PTX were administered with IP PTX on days 1, 8, and 15, followed by 1 week of rest. This treatment was repeated until intolerable toxicity, disease progression, or conversion surgery occurred. RFS and OS were 6 and 14.5 months, respectively. The response ratio and DCR were 50% and 95%, and the 1-year survival rate was 61%, with 39% of patients showing a change from positive to negative cytology. This study also showed that OS for patients with conversion surgery was significantly longer than for those without conversion surgery, though results were not reached versus 12.4 months [4].

However, the former study enrolled patients without distant metastasis, excluding the ovaries and massive ascites, while the latter study enrolled patients without distant metastasis. This suggests that patient selection is crucial for success of IP and IV chemotherapy in pancreatic cancer. Continued efforts are necessary to conduct well-designed randomized clinical trials to confirm the efficacy of IP and IV chemotherapy for PMs in pancreatic cancer. A phase III study is currently in progress. This trial evaluates an intraperitoneal IP PTXregimen for PDAC with PMs. The study compares combined IV and IP PTX with S-1 against the standard systemic regimen of IV GEM plus IV nab-PTX. In the investigational S1-PTX group, IV PTX is administered at 50 mg/m^2^ and IP at 20 mg/m^2^ on days 1 and 8 of a 21-day cycle through an implanted peritoneal access port, while S-1 is given orally twice daily on days 1–14. In the comparison group with GEM plus nab-PTX group, nab-PTX 125 mg/m^2^ and GEM 1000 mg/m^2^ are administered on days 1, 8, and 15 of a 28-day cycle. Treatment in both groups continues until disease progression, unacceptable toxicity, conversion surgery, or patient withdrawal. The primary endpoint is OS, and secondary endpoints include response rate, PFS, conversion of peritoneal washing cytology, symptom improvement, tumor marker reductions, and the rate of conversion surgery. A total of 180 patients with PMs will be enrolled across 30 institutions, with randomization stratified by institution. This trial enables a direct comparison between an IP PTX–based regimen and standard systemic chemotherapy in PDAC with PMs [71].

### 6.3. Gastric Cancer

PMs are common and formidable in GC. Estimated synchronous incidence of PMs is about 10–21% at initial diagnosis and median overall survival is typically less than one year under treatment with systemic chemotherapy [72]. Nationwide cohort data confirm high incidence and poor outcomes for synchronous PM of GC in routine practice [73]. Even after curative gastrectomy, peritoneal relapse occurs with a probability of 40% and remains a dominant failure pattern, tightly associated with inferior survival [74]. As mentioned above, the modest activity of systemic therapy against PMs reflects the blood–peritoneal barrier and unique peritoneal tumor microenvironment. Previous knowledge motivates researchers to investigate efficacy of peritoneum-directed delivery.

Among IPC strategies, the combination of CRS and HIPEC has the longest clinical track record in treating PMs from GC. Numerous studies, including RCTs, have evaluated the therapeutic value of adding HIPEC to CRS. As these findings have been comprehensively reviewed elsewhere, with most analyses suggesting partial survival benefits without increased morbidity [75,76,77], here we briefly revisit the results of the major RCTs. In 2011, the first prospective RCT from China enrolled 68 GC patients who had synchronous or metachronous PMs [78]. The HIPEC regimen comprised cisplatin (CDDP) 120 mg and mitomycin C (MMC) 30 mg dissolved in 6000 mL of saline at 43 °C, perfused for 60–90 min. The study demonstrated a statistically significant improvement in median overall survival (OS) with CRS + HIPEC compared to CRS alone (11.0 vs. 6.5 months; *p* = 0.046). In 2019, a phase III multicenter RCT (CYTO-CHIP study) using inverse probability of treatment weighting analysis showed that CRS + HIPEC significantly improved OS and relapse-free survival (RFS) compared with CRS alone in 277 patients (18.8 vs. 12.1 months and 13.6 vs. 7.8 months, respectively), particularly in those with low peritoneal cancer index (PCI) and complete cytoreduction. The incidence of grade 3–5 postoperative complications was comparable between groups [23]. More recently, the phase III GASTRIPEC-I trial (2024) investigated whether CRS + HIPEC improves outcomes in GC patients with PMs [5]. A total of 105 patients from 23 German centers were randomized to receive either CRS alone or CRS + HIPEC. The HIPEC regimen consisted of MMC 15 mg/m^2^ and CDDP 75 mg/m^2^ perfused at 42 °C for 60 min. Median OS did not differ significantly between the two groups (14.9 months for both; *p* = 0.16), but CRS + HIPEC significantly prolonged progression-free survival (PFS; 7.1 vs. 3.5 months, *p* = 0.047) and metastasis-free survival (MFS; 10.2 vs. 9.2 months, *p* = 0.028). Rates of severe postoperative complications were also similar (43.6% vs. 38.1%; *p* = 0.79). Notably, subgroup analysis revealed that patients who achieved complete cytoreduction (CC-0) derived a significant OS benefit from HIPEC (*p* = 0.043), underscoring the critical importance of complete surgery. No OS benefit was observed in cases with incomplete cytoreduction. Although the GASTRIPEC-I trial concluded that HIPEC does not extend OS in the overall population, its findings indicate a potential benefit for patients achieving complete CRS, with improved PFS and MFS. A major strength of this trial was the standardized treatment protocol across participating centers, in contrast to earlier studies. Collectively, these findings further support the therapeutic rationale and potential efficacy of HIPEC for GC patients with PMs.

However, the most critical limitation of these HIPEC studies is the absence of direct comparison with outcomes from patients receiving systemic chemotherapy, which remains the standard treatment for unresectable or metastatic gastric cancer. From this perspective, catheter-based intraperitoneal chemotherapy (IPC) combined with systemic chemotherapy was developed in Japan. A phase II clinical trial was conducted to evaluate efficacy and safety of combined intraperitoneal paclitaxel (IP PTX) and intravenous paclitaxel (IV PTX) plus S-1 in patients with macroscopic PMs or positive peritoneal cytology [79]. Forty patients were enrolled, including 21 with macroscopic PMs, 13 with peritoneal recurrence, and 6 with cytology-positive disease only. Treatment consisted of daily oral S-1 (80 mg/m^2^) for 14 consecutive days followed by a 7-day rest, combined with weekly IV PTX (50 mg/m^2^) and IP PTX (20 mg/m^2^) administered on days 1 and 8 of a 21-day cycle through an implanted peritoneal port. The 1-year overall survival (OS) rate was 78% (95% CI: 65–90%), and the median OS reached 22.5 months. Among 18 patients with measurable lesions, the overall response rate was 56%. Malignant ascites disappeared or decreased in 13 of 21 cases (62%), and peritoneal cytology converted to negative in 24 of 28 cases (86%). Grade 3–4 toxicities included neutropenia (38%), leukopenia (18%), and anemia (10%), whereas no treatment-related deaths or severe IP-specific complications such as peritonitis or catheter infection were observed. 

Another phase II trial targeted a selected cohort of GC patients with macroscopic PMs [80]. A total of 35 patients with advanced or recurrent GC presenting with PMs were enrolled. The 1-year overall survival (OS) rate was 77.1%, and the median OS was 17.6 months (95% CI: 13.4–not reached), with a 2-year OS rate of 44.8%. Among seven patients with measurable peritoneal lesions, five (71%) achieved partial responses. Malignant ascites decreased or disappeared in 15 of 22 patients (68%), and peritoneal cytology converted to negative in 28 of 29 patients (97%). Grade 3–4 hematologic toxicities included neutropenia (34%), leukopenia (23%), and anemia (9%). Notably, no cases of abdominal pain, infusion-related toxicity, or treatment-related death were reported. The two preceding phase II studies paved the way for a phase III RCT (PHOENIX-GC), which evaluated whether combined intraperitoneal (IP) and intravenous (IV) paclitaxel (PTX) plus S-1 (the IP regimen) could improve outcomes compared with cisplatin (CDDP) plus S-1 (the SP regimen), the standard first-line therapy for GC patients with PMs [1]. A total of 183 patients from 20 Japanese centers were randomized to either the IP or SP group. The former received the same regimen as that described by Ishigami et al. (2010) [79], whereas the SP group received CDDP (60 mg/m^2^) on day 8 and daily oral S-1 (80 mg/m^2^) for 21 days of a 5-week cycle. After excluding ineligible patients, 164 were included in the final analysis. Baseline characteristics were generally well balanced, except for a higher incidence of ascites in the IP group. Median overall survival (OS) was 17.7 months in the IP group and 15.2 months in the SP group (hazard ratio [HR], 0.72; *p* = 0.08). Three-year OS rates were 21.9% and 6.0%, respectively. In a sensitivity analysis adjusted for the presence of ascites, the HR improved to 0.59 (*p* = 0.008), indicating a significant survival benefit associated with IP therapy. The IP group also demonstrated higher rates of ascites reduction and cytology conversion from positive to negative (76% vs. 33%). In conclusion, although the PHOENIX-GC trial did not meet its primary endpoint of statistical superiority for OS, exploratory analyses revealed clinically meaningful benefits, particularly in patients with ascites. These findings support IP-PTX as a promising therapeutic option for GC patients with unresectable PMs.

Several RCTs are currently underway worldwide to validate clinical benefits of this approach. Various clinical studies have explored IPC with PTX in GC patients with PMs, often combined with systemic therapy, yielding encouraging outcomes. For instance, a Japanese multicenter phase II trial evaluated IP PTX plus S-1 and CDDP in 53 patients with PMs. This regimen (IP PTX 20 mg/m^2^ on days 1, 8, 22 every 5 weeks, with oral S-1 and IV CDDP) achieved a 1-year OS of 73.6% and median OS of 19.4 months. The 1-year PFS was 49.6%. Toxicity was substantial, but manageable, with grade 3–4 hematologic and non-hematologic adverse events in 43% and 47% of patients, including most commonly neutropenia (25%) and anemia (30%), along with diarrhea (13%) and anorexia (17%), respectively. Four patients (7.5%) experienced catheter-related complications and one treatment-related death occurred. Nonetheless, the authors concluded that IP PTX combined with S-1 plus CDDP is attractive and tolerable in this setting [81]. Similarly, a Korean phase II trial investigated combination therapy with IP PTX plus systemic S-1 and L-OHP in advanced or recurrent GC with PMs. In that prospective multicenter study, patients received IP PTX 80 mg/m^2^ on days 1 and 8, S-1 80 mg/m^2^ daily on days 1–14 and L-OHP 100 mg/m^2^ on day 1, repeated every 3 weeks. Among 24 evaluable patients, the primary endpoint of 6 months PFS was 82.6%. 1-year PFS and OS reached 69.6% and 76.9%, respectively. The response rate was 41.7% and notable grade 3–4 hematologic toxicities were neutropenia (41.7% of patients) and leukopenia (20.8%) with grade 3 diarrhea as the main non-hematologic toxicity. Approximately 12.5% of patients had catheter-related complications. The study concluded that IP PTX combined with systemic S-1 and L-OHP shows promising efficacy and a tolerable safety profile [82]. Another combination regimen has been tested in Singapore. Chia et al. reported a phase II trial of IP PTX plus systemic capecitabine and L-OHP in GC patients with PMs. Notably, 95% of patients in this study had PMs visible on CT or laparoscopy and more than 90% had poorly differentiated tumors. Despite this heavy tumor burden, the median OS reached 14.6 months and importantly, a subset of patients who underwent conversion surgery after chemotherapy achieved a prolonged OS of 24.2 months, which is comparable to those with aggressive surgery in similar populations. The authors noted the absence of a PCI score as a limitation, but the findings demonstrated that IP PTX combined with systemic capecitabine and L-OHP may improve prognosis of GC patients with PMs [47]. In the West, a prospective phase II trial called STOPGAP is also underway to evaluate a sequential strategy of systemic therapy followed by IP PTX for GC patients with PMs [83]. The STOPGAP trial enrolls patients with positive peritoneal cytology or gross peritoneal metastases who first receive 3 months of standard systemic chemotherapy. Those without disease progression then proceed to IP PTX combined with systemic chemotherapy with PTX and 5-FU administered on days 1 and 8 of a 21-day cycle for four cycles. Diagnostic laparoscopy is performed before and after IP treatment to assess PCI score and patients who achieved PCI ≤ 10 with resectable disease might undergo CRS with HIPEC. The primary endpoint of STOPGAP is 1-year PFS with secondary endpoints including OS and quality of life. This trial seeks to determine whether a sequential multimodal approach could improve outcomes, given that the prior Asian PHOENIX-GC trial suggested survival benefits from IP PTX therapy, despite not achieving a statistically significant difference in OS. Results from ongoing trials are eagerly awaited to clarify the role of IPC in GC with PMs and to potentially integrate IP PTX into standard treatment protocols. To build on the success of some phase II studies, the DRAGON-01 trial is an ongoing phase III RCT in China [84]. The Dragon 01 trial already reported encouraging results at the American Society of Clinical Oncology Annual Meeting 2025. In this multicenter phase III RCT, 222 GC patients with PMs were randomized to receive either IP PTX combined with systemic chemotherapy (IV PTX plus S-1) or systemic therapy alone. The IP group demonstrated a significant improvement in OS (19.4 vs. 13.9 months; *p* = 0.054) including 1 year OS rate (69.6% vs. 54.1%), 2 years (37.2% vs. 20.3%), 3 years (24.3% vs. 12.2%), and 5 years (11.4% vs. 7.9%) with manageable toxicity profiles and no increase in treatment-related mortality. These outcomes further strengthen the evidence supporting IP-based combination therapy as a feasible and effective strategy for improving prognosis of GC patients with PMs. Further accumulation of clinical experience is expected to help identify optimal protocols and patient selection criteria that might extend survival benefits of catheter-based IPC using PTX combined with systemic chemotherapy.

### 6.4. Colorectal Cancer

Colorectal cancer (CRC) accounts for approximately 10.2% of all newly diagnosed cancers worldwide, with about 1.8 million new cases, making it the third most common cancer. CRC is also the second leading cause of cancer-related death, although some risk factors are suggested, such as obesity, alcohol, and processed meat [85]. As for frequency of PMs from CRC, a population-based cohort study was conducted in Sweden and 11,124 patients diagnosed with CRC between 1995 and 2007 were analyzed. A total of 924 patients (8.3%) developed PMs, including 477 patients (4.3%) with synchronous and 447 patients (4.2%) with metachronous PMs [86]. PMs represent one of the most unfavorable metastatic patterns in CRC, being strongly associated with poor prognosis. In 2013, a large institutional registry of 2406 CRC patients reported that 10.6% developed PMs including 4.8% synchronous and 5.9% metachronous cases whose data are generally consistent with the aforementioned study. Outcomes were dismal with a median OS of 17.9 months and 5-year survival rates of 8.1% for synchronous and 25.4% for metachronous PMs. Independent predictors of PMs, T4 stage, nodal metastases, left-sided or appendiceal origin, non-CRS, and age > 61 years, are associated with lower risk [87]. Moreover, a Dutch nationwide study of 1235 CRC patients with synchronous PMs who received only palliative systemic therapy with L-OHP showed that addition of bevacizumab (Bev) significantly prolonged median OS from 7.5 to 11.0 months (HR 0.7, 95% CI 0.64–0.83). This benefit was observed both in isolated PMs and in cases with concurrent extraperitoneal metastases, suggesting that addition of Bev might modestly improve outcomes, but does not overcome the poor prognosis of this disease [88]. Furthermore, analysis of 10,553 patients by Analysis and Research in Cancers of the Digestive System demonstrated that PMs independently predicted poor survival compared with solitary liver or lung metastasis (adjusted HR 0.75, *p* = 0.003) [89]. Even isolated PMs showed significantly worse outcomes, confirming that PMs represent a treatment-resistant condition.

Overall, CRC with PMs remains a clinical challenge with limited survival benefit from systemic chemotherapy, emphasizing the need for improved comprehensive multimodal or locoregional treatment strategies. Accordingly, over the past several decades, numerous studies have investigated the efficacy of CRS combined with HIPEC in patients with colorectal cancer (CRC) presenting with peritoneal metastases (PMs). In the first phase III RCT conducted in the Netherlands, Verwaal et al. compared CRS plus HIPEC using mitomycin C with systemic chemotherapy alone in 105 patients with PMs [3]. Those results demonstrated significantly prolonged median overall survival (OS) in the CRS + HIPEC group compared with the systemic chemotherapy group (22.3 months vs. 12.6 months, *p* = 0.032). However, the treatment-related mortality rate in the CRS + HIPEC group was 8%, primarily due to bowel leakage and sepsis. Subgroup analysis revealed that patients with limited peritoneal disease (involving five or fewer of the seven abdominal regions) who achieved complete macroscopic cytoreduction experienced markedly better outcomes, with a median survival exceeding 29 months, whereas those with extensive disease (six to seven regions) had a median survival of only 5.4 months (*p* < 0.0001). Collectively, this pivotal study indicated that CRS + HIPEC confers a significant survival advantage over systemic chemotherapy alone, although the benefit is largely restricted to patients with limited peritoneal involvement in whom complete macroscopic resection is achievable. Subsequent comparative studies have consistently corroborated these findings. Franko et al. demonstrated that patients who received HIPEC with mitomycin C (MMC) in combination with systemic chemotherapy (oxaliplatin- or irinotecan-based regimens, with or without targeted agents) achieved a median overall survival (OS) of 34.7 months, compared with 16.8 months in those treated with systemic therapy alone (*p* < 0.001). Addition of targeted agents further prolonged survival (30.3 vs. 19.7 months, *p* = 0.02) [25]. Similarly, Elias et al. reported excellent long-term outcomes, with a median OS of 33.4 months and a 5-year survival rate of 29.7% [90] Cashin et al. also demonstrated an improved 2-year overall survival of 54% in the CRS + HIPEC group when compared to 24% in the control group with systemic chemotherapy alone [91]. In a large French multicenter study of 523 patients, complete CRS followed by HIPEC using MMC or oxaliplatin achieved a median OS of 30.1 months and a 5-year survival rate of 27% [92] Complete cytoreduction had a highly significant impact on prognosis (*p* < 0.001), whereas neither histologic grade nor the presence of synchronous liver metastasis adversely affected outcomes when complete resection was achieved. Likewise, another Dutch study involving 660 cases reported a median OS of 33 months and a recurrence-free survival (RFS) of 15 months [93].

A meta-analysis by Mirnezami et al. confirmed that addition of CRS + HIPEC to systemic chemotherapy significantly improved both 2- and 5-year survival rates, with odds ratios (ORs) of 2.78 and 4.07, respectively [26]. Collectively, these studies provide robust evidence that CRS combined with HIPEC using MMC or oxaliplatin, together with systemic chemotherapy, confers a statistically significant survival advantage in carefully selected patients with limited peritoneal metastases who achieve complete cytoreduction. However, whether observed benefits reflected the use of CRS or HIPEC remained uncertain. To address this critical question, the multicenter randomized controlled PRODIGE 7 trial was undertaken as the first large-scale study specifically designed to evaluate the additive impact of HIPEC in this setting. After achieving complete or near-complete cytoreduction (CC-0/1), 267 patients were randomly assigned to either the HIPEC or non-HIPEC arm. All patients received preoperative systemic chemotherapy, and oxaliplatin was used as the intraperitoneal agent for HIPEC. The median OS was 41.7 months in the HIPEC group and 41.2 months in the non-HIPEC group, showing no significant difference between the two. Subgroup analysis revealed that patients with an intermediate peritoneal cancer index (PCI = 11–15) derived greater benefit from HIPEC. Although the study did not demonstrate a statistically significant OS advantage for HIPEC, CRS alone achieved excellent outcomes, with a 5-year survival rate of 49% [94]. Taken together, these findings support CRS combined with HIPEC as an effective locoregional therapeutic strategy for PMs from CRC. However, the inconsistent evidence regarding its efficacy and its potential for increased toxicity have limited widespread implementation in clinical practice. From this standpoint, PIPAC has emerged as a promising alternative to HIPEC. While HIPEC exerts its antitumor effects primarily through hyperthermia, PIPAC enhances the cytotoxic potential of chemotherapy by applying intraperitoneal pressure [32].

A key advantage of PIPAC is its feasibility and repeatability in most patients, as it can be performed safely via minimally invasive laparoscopic access to the peritoneal cavity [95]. Some reviews have demonstrated that PIPAC is feasible, safe, and well-tolerated by patients with peritoneal metastases of colorectal origin. Sleiman et al. analyzed 949 patients in 11 studies and found that most patients completed the planned 2–3 PIPAC cycles. In that cohort, the median OS ranged from 8 to 37.8 months [96]. Similarly, Lurvink et al. reported a median OS of 15–27 months in colorectal patients treated with oxaliplatin-PIPAC [97]. These figures are consistent with Alyami et al. who estimated a median OS of 16 months in CRC with PMs [33]. Objective tumor responses are generally high. In the systematic analysis, 50% of patients achieved a complete histopathologic response and 29% had major regression of PMs after PIPAC [97]. Assessments using computed tomography are concordant. For example, 28% of patients showed measurable tumor shrinkage after PIPAC, whereas 36% had stable disease. Cytological responses were also observed. Approximately, one third of patients converted from positive to negative peritoneal cytology during treatment [97]. These results indicate significant antitumor activity. Adverse events and tolerability were relatively favorable in all reviews. In the Sleiman et al. review, most postoperative complications were grade 1–2 and no intraoperative complications were reported [96]. The incidence of CTCAE grade ≥ 3 event was only 12–15% of PIPAC [33]. In fact, Lurvink et al. found that only 4 of 21 patients (19%) experienced any grade 3 toxicity of abdominal pain, with no grade 4–5 events. Importantly, no study reported PIPAC-related deaths. Side effects of grade 1–2 were normal. These reviews reported that abdominal pain and nausea occurred in one-third of patients [97]. Notably, two studies reported that quality of life was preserved or slightly improved during PIPAC [33,96]. Overall, these data show that PIPAC produces substantial tumor control and survival benefit with minimal high-grade toxicity in CRC with PMs. In summary, current evidence showing good survival, strong treatment responses and low complication rates suggests that PIPAC is a practical, safe, and well-tolerated treatment option for CRC patients with PMs. In addition, catheter-based IP-PTX therapy has been employed in Japan for treatment of PMs from CRC. The iPac-02 study was a single-arm, multicenter phase II trial enrolling 38 patients with isolated, unresectable colorectal peritoneal carcinomatosis to evaluate weekly IP-PTX (20 mg/m^2^) combined with FOLFOX- or CAPOX-bevacizumab. The trial was designed with response rate as the primary endpoint and progression-free survival, overall survival, peritoneal cancer index improvement, conversion to negative peritoneal cytology, and safety as secondary endpoints. This study was initiated on the basis of favorable safety and preliminary efficacy observed in the preceding phase I trial, which showed a response rate of 25%, PCI improvement in 50% of patients, complete cytology conversion, a progression-free survival of 8.8 months, and a median survival of 29.3 months. The iPac-02 trial aims to prospectively validate these findings and more clearly define the clinical utility of IP-PTX for colorectal peritoneal metastases [98]. Nevertheless, the definitive impact on survival outcomes and quality of life of these approaches remains to be established, as robust comparative studies in large cohorts are still lacking.

### 6.5. Pseudomyxoma Peritonei

Peritoneal pseudomyxoma (PMP) is a rare clinical entity affecting a few individuals per million annually. It is characterized by mucinous ascites of variable cellularity and malignant potential. In nearly 90% of cases, PMP originates from ruptured appendiceal mucinous neoplasms (LAMN). Prognosis largely depends on the histologic grade and peritoneal dissemination, as defined by the 2019 WHO classification. Accumulation of mucin in the peritoneal cavity can lead to severe abdominal distension and bowel dysfunction, for which palliative debulking may improve both quality of life and survival. Currently available systemic therapies have limited efficacy in altering the natural course of PMP. Consequently, the global standard of care for this disease remains complete CRS followed by HIPEC. Although no RCT has directly demonstrated the benefit of HIPEC over CRS alone, evidence from large retrospective cohorts supports its therapeutic value. An international registry analysis by the Peritoneal Surface Oncology Group International (PSOGI), including 1924 patients, showed that CRS combined with HIPEC was associated with significantly improved survival compared with CRS alone. Concrete, 5-year OS was 57.8% with CRS + HIPEC versus 46.2% with CRS alone. After multivariate analysis, HIPEC remained associated with improved survival (HR 0.65, *p* = 0.001). Rates of severe adverse events, reoperation, and 30- or 90-day mortality did not significantly differ between groups. These findings indicate that adding HIPEC to complete CRS provides a measurable survival benefit without increasing major postoperative risk [99]. Early postoperative intraperitoneal chemotherapy (EPIC) has also emerged as a potentially beneficial adjunct. First introduced by Sugarbaker to eradicate microscopic residual disease after CRS [100], EPIC typically involves intraperitoneal administration of 5-fluorouracil for five consecutive postoperative days, achieving high local concentrations with minimal systemic toxicity. While early comparative studies favored HIPEC for its efficacy and lower complication rates, subsequent evidence suggests that combining HIPEC and EPIC may further improve long-term outcomes. In a large international registry of 2298 patients with appendiceal PMP treated with CRS and HIPEC, Chua et al. demonstrated a strong prognostic influence of intraperitoneal chemotherapy (IPC). HIPEC, using mitomycin C (10–12.5 mg/m^2^) or oxaliplatin (460 mg/m^2^), was performed after complete cytoreduction (<2.5 mm residual disease), followed by EPIC with intraperitoneal fluorouracil (650 mg/m^2^) on postoperative days 1–5. Patients treated with CRS + HIPEC achieved 5- and 10-year overall survival (OS) rates of 78% and 68%, respectively, compared with 40% and 27% in those without HIPEC (*p* < 0.001). Addition of EPIC further improved outcomes, with 5- and 10-year OS rates of 84% and 73% versus 69% and 57% without EPIC (*p* < 0.001). Multivariate analysis identified HIPEC as an independent favorable factor for progression-free survival (HR 0.65, *p* = 0.03), whereas incomplete CRS, high peritoneal cancer index (PCI), postoperative complications, and high-grade histology predicted poor outcomes. Notably, EPIC, although selectively used in clinically stable patients, did not increase severe morbidity while conferring an additional survival benefit [27]. Similarly, Huang et al. (2017) reported improved 5-year OS for combined HIPEC and EPIC therapy in both low-grade appendiceal mucinous neoplasms (93.0% vs. 64.5%, *p* = 0.001) and peritoneal mucinous carcinomatosis (62.3% vs. 30.5%, *p* = 0.002), without an associated rise in morbidity [101]. However, the retrospective nature and potential selection bias in these studies warrant cautious interpretation. Collectively, current evidence suggests that while HIPEC remains the cornerstone of PMP management, EPIC may provide an additional survival advantage when appropriately combined with HIPEC in selected patients.

However, patients with extensive or unresectable disease remain ineligible for initial CRS, and effective therapeutic strategies for this subgroup have not yet been established. In a prospective phase II study, Prabhu et al. investigated the feasibility of neoadjuvant IPC in PMP patients who were initially unsuitable for CRS + HIPEC [102]. Twenty-seven patients, predominantly with appendiceal origin (85.2%), underwent laparoscopic HIPEC using oxaliplatin (L-OHP 200 mg/m^2^) followed by intraperitoneal administration of docetaxel (40 mg/m^2^) and cisplatin (CDDP 40 mg/m^2^) in combination with oral S-1 for 14 days every 3 weeks. After a median of five treatment cycles, 22 patients (81.5%) became eligible for CRS + HIPEC, with complete cytoreduction (CC-0/1) achieved in 54.5% of them. A major pathological response was observed in 91% of patients, accompanied by a 28% reduction in mean carcinoembryonic antigen (CEA) levels and a mean decrease in peritoneal cancer index (PCI) of 2.6. Furthermore, positive peritoneal cytology converted to negative in 69% of patients. Rates of major morbidity and mortality were 13.6% and 4.5%, respectively, indicating acceptable safety. These results suggest that neoadjuvant IPC may effectively downstage disease and broaden surgical eligibility in patients with advanced PMP. Although survival outcomes remain preliminary, this pilot study provides a strong rationale for further validation.

Overall, while CRS combined with IPC has yielded encouraging clinical results, a standardized treatment regimen for PMP has not yet been defined. Large-scale prospective studies, including RCTs, are warranted to establish the optimal therapeutic strategy and to confirm the survival benefit of neoadjuvant IPC in this rare disease.

### 6.6. Hepatocellular Carcinoma

PMs from hepatocellular carcinoma (HCC) are uncommon, but emerging evidence suggests that aggressive treatment can benefit selected patients. Notably, Mehta et al. reported a multicenter retrospective study of 21 patients undergoing CRS + HIPEC for HCC PMs. The median OS was 46.7 months, with 3- and 5-year OS rates of approximately 89% and 49%, respectively. Importantly, patients who achieved complete cytoreduction had significantly better outcomes. The median OS was not reached in this group, whereas it was only 6 months for those with incomplete resection [103]. Another recent study of 10 patients at single institution in China demonstrated similarly encouraging favorable survival with CRS + HIPEC. In this study, 1- and 3-year OS rates were both 89%, although the 5-year OS rate declined to 21%. The median PFS was only 5 months, indicating that while early recurrences are common, a subset of patients can survive more than 3–5 years after CRS + HIPEC, using cisplatin 120 mg and docetaxel 120 mg [104]. These findings underscore the potential of CRS + HIPEC to extend survival in HCC patients with PMs, provided that a complete tumor resection is achievable. Moreover, in situations where HCC carries a high risk of PMs, such as tumor rupture, HIPEC may serve a prophylactic purpose. Li et al. recently compared outcomes in 77 patients with spontaneously ruptured HCC who underwent liver resection with or without HIPEC consisting of platinum (CDDP 75–100 mg/m^2^ or L-OHP 100–150 mg/m^2^) + pyrimidine (GEM 800–1000 mg/m2 or 5-FU 1000–1500 mg). The HIPEC group showed significantly higher 3 year OS (74% vs. 44%) and RFS (55% vs. 24%) without increasing perioperative morbidity [105]. This suggests that HIPEC can be safely integrated after CRS to improve disease control in high-risk HCC cases. Overall, while no prospective trials exist yet, accumulating clinical data support considering CRS + HIPEC for carefully selected HCC patients with PMs to enable long survival.

## 7. Future Directions

Major clinical trials for each tumor type presented above are summarized in Table 2, regarding administered drugs and outcomes.

Adverse events are summarized in Table 3. 

Emerging therapeutic strategies for PMs focus on improving pharmacologic synergy between IP and systemic chemotherapy while addressing biological limitations that reduce treatment efficacy. The most promising direction lies in developing bidirectional regimens that target both the peritoneal cavity and systemic circulation, thereby overcoming anatomical and physiological constraints imposed by the peritoneum–plasma barrier. Combination approaches using sequential or simultaneous IP and systemic drug administration are now being refined to maximize cytotoxic exposure while minimizing systemic toxicity. Importantly, two RCTs in Japan clearly showed that IP combined with systemic chemotherapy achieves better control of prognosis and ascites, which underscores the pharmacokinetic advantage of IP administration and its potential to act synergistically with systemic chemotherapy in eradicating residual IP disease [1,2].

From a pharmacological standpoint, innovations in drug delivery systems are also under investigation to maximize local drug exposure, improve uneven drug coverage, and limit systemic toxicity. Nanoparticle-based delivery systems are emerging as a transformative approach to improve IP pharmacokinetics. Engineered nanocarriers such as liposomes and polymeric nanoparticles are designed to enhance efficacy against tumors and retention in the peritoneal cavity by exploiting enhanced permeability and retention by tumor vasculature. For example, ultra–high drug loading PTX nanoparticles for peritoneal mesothelioma achieved higher PTX concentrations than standard formulations and longer survival in mouse models with only two IP doses. Such findings suggest that tumors deemed chemoresistant may respond once drug delivery is optimized [106].

Parallel strategies involve “smart nanoparticles” that release active ingredients in response to TME, such as hydrogen ion exponent or redox conditions, concentrating effects of drugs at tumor sites and minimizing off target toxicity. This targeted release mechanism may also prove valuable in the peritoneal cavity because releasing drugs throughout a large area induces tissue irritation and causes local adhesions.

Furthermore, optimal timing between IP and systemic administration remains to be determined. Although few studies have addressed this issue, Tamura et al. investigated it in a mouse model by administering IV CBDCA either on the same day, 1 day, or 4 days after IP PTX and measuring CBDCA concentrations of PMs. Interestingly, the group receiving CBDCA 4 days after IPC showed significantly higher CBDCA levels. However, it remains unclear which interval is optimal because administration 7 or 14 days later or even longer may yield better results. Moreover, because the tested drugs were limited, further investigation is warranted. Once optimal timing and drug combinations are identified, this approach could be readily implemented with currently available drugs and might provide an easily applicable clinical strategy [107].

Finally, IPC also affects peritoneal tumor TME [108]. Some studies demonstrate that IPC remodels the tumor stroma and disrupts the extracellular matrix (ECM), breaking physical barriers that restrict chemotherapeutic penetration [109,110]. This includes altering the activity of stromal cells such as cancer-associated fibroblasts, which reduces secretion of protective matrix proteins and pro-survival factors that shield tumor cells from chemotherapy [109,111]. Moreover, HIPEC provokes local immune activation by inducing immunogenic cell death and associated danger signals. This immune stimulation is evidenced by increased infiltration and activation of cytotoxic T cells and macrophages in peritoneal tumors, effectively converting an immunosuppressive TME to anti-tumor TME [112]. In addition, high concentrations of IP overcome drug resistance mechanisms because saturating efflux pumps and providing sustained drug exposure improve drug accumulation in chemoresistant tumor cell populations [111]. Notably, hyperthermia used in HIPEC could augment chemosensitivity by facilitating immune-mediated elimination of residual cancer stem like cells [112]. Collectively, these effects indicate that functions of IPC not only kill tumor cells directly, but also reshape the peritoneal metastatic niche through stromal remodeling, ECM disruption, and immune activation, which may mitigate TME-driven drug resistance and improve prognosis [113].

Thus, despite the encouraging results achieved to date, considerable room for improvement remains in various aspects of combined IP and systemic chemotherapy. Continued translational research and well-designed clinical trials will be essential to further refine treatment strategies and to establish more definitive evidence of clinical benefit.

## 8. Limitations

Throughout this review, we distinguished between findings from early phase trials and those from more rigorous late phase trials, in order to avoid overstating conclusions. In particular, many included studies were Phase II single arm trials, which lack a control group and therefore provide a lower level of evidence. Results from such studies are inherently exploratory and subject to selection bias or confounding. In contrast, Phase III RCTs offer higher evidentiary weight due to their comparative design and larger sample sizes. We have explicitly identified phases and designs of trials when discussing outcomes, and we interpreted those data with appropriate caution. Promising efficacy signals observed in Phase II studies were treated as hypothesis-generating rather than as definitive. Consequently, our conclusions have been understated to reflect the tentative nature of non-randomized evidence. Therefore, we avoid generalizing single cohort results to broad clinical practice without confirmation from Phase III trials. This tempered interpretive stance ensures that recommendations are appropriate to the strength of evidence, aligning with the principle that preliminary findings require validation before altering standards of care. By acknowledging these differences in evidence level, we ensure that the review’s clinical inferences remain balanced and credible.

Moreover, we note that a substantial portion of the clinical data on IPC for PMs originates from Japan and South Korea. Many of the pivotal trials and case series in this field have been conducted in Asian populations and patients enrolled in these studies have been predominantly of Asian ethnicity. This regional skew in the literature raises concerns about external validity. Results that showed efficacy and safety in Asian patient cohorts may not be fully generalizable to Western populations or other ethnic groups, given potential differences in tumor biology, genetic factors, and treatment practices across regions. We therefore explicitly acknowledge this limitation. Outcomes reported in Japanese and Korean studies are informative, but may reflect population-specific factors. The geographic and ethnic bias in these data means that overall conclusions of our review should be applied with caution in other contexts. Ongoing and future trials in Europe, North America, and other regions will be crucial to determine whether benefits observed with IPC + systemic chemotherapy in Asian trials can be reproduced.

## 9. Conclusions

**Figure 2:**
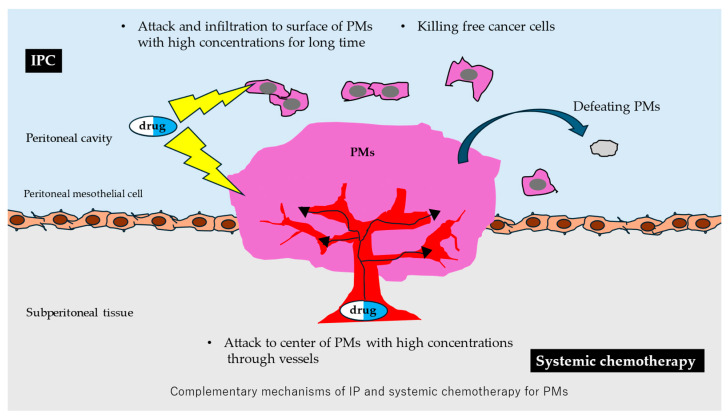
Schematic overview of the therapeutic advantages of combined IPC and systemic chemotherapy for PMs.

Combining IP and systemic chemotherapy represents a promising therapeutic strategy for peritoneal metastases and has the potential to improve prognosis in selected patients (Figure 2). With further optimization and clinical validation, this approach may ultimately become an important component of future multimodal cancer treatment strategies.

## Figures and Tables

**Table 1:** Advantages and Disadvantages of IPC for PMs.

Advantages	Disadvantages
Directly targets PMs and free intraperitoneal cancer cells with high local drug concentrations.	Requires invasive procedures (port-based IPC requires surgical port placement or HIPEC is one-time during major surgery).
Prolonged local drug exposure due to limited systemic absorption, leading to reduced systemic toxicity.	Needs specialized equipment and expertise, making it resource-intensive and high-cost.
Deeper penetration of chemotherapy into PMs.	High risk of severe side effects as for hematologic or non-hematologic toxicity).
Implantation of IP port enable repeatable administration and outpatient treatment.	Risk of port-related complications (infection, catheter blockage, leakage).
IPC can convert some patients to surgery.	Limited evidence from large trials and unclear optimal patient selection.
HIPEC further enhances drug penetration and tumor cell kill, achieving high local efficacy.	Does not favorably affect disease outside the peritoneal cavity.

**Table 2:** Clinical Trials Summary by Cancer Type. n.s: not significant.

Tumor Type	Author, Year	Study, Design	Treatment Regimen	Outcomes	Remarks
Ovarian	Alberts et al. (1996) [57]	Phase III RCT	IV CDDP 100 mg/m^2^ + IV CPA 600 mg/m^2^ vs. IP CDDP 100 mg/m^2^ + IV CPA 600 mg/m^2^	OS: 41 (IV group) vs. 49 months (IP group) (*p* = 0.02)	IP group has significantly longer OS.
	Markman et al. (1998) [58]	Phase III RCT	IV PTX 135 mg/m^2^ + IV CDDP 75 mg/m^2^ vs. IV CBDCA (AUC9) + IV PTX 135 mg/m^2^ + IP CDDP 100 mg/m^2^	PFS: 22 (IV group) vs. 28 months (IP group) (*p* = 0.01); OS: 52 (IV group) vs. 63 months (IP group) (*p* = 0.05)	IPC shows improved PFS and a trend toward OS benefit.
	Armstrong et al. (2006) [59]	Phase III RCT	IV PTX 135 mg/m^2^ + IV CDDP 75 mg/m^2^ vs. IV PTX 135 mg/m^2^ + IP CDDP 100 mg/m^2^ + IP PTX 60 mg/m^2^	PFS: 18.3 (IV group) vs. 23.8 months (IP group) (*p* = 0.05); OS: 49.7 (IV group) vs. 65.6 months (IP group) (*p* = 0.03)	Markedly better PFS/OS with IP, but <50% completed 6 cycles due to toxicity
	Nagao et al. (2020) [2]	Phase II/III RCT	IV PTX 80 mg/m^2^ (days 1, 8, 15) + IV CBDCA AUC6 vs. IV PTX 80 mg/m^2^ (days 1, 8, 15) + IP CBDCA AUC6	PFS: 20.7 (IV group) vs. 23.5 months (*p* = 0.04); OS: 67.0 (IV group) vs. 64.9 months (IP group) (*p* = n.s)	Significant PFS benefit with IP
Pancreatic	Satoi et al. (2017) [70]	Phase II	IV PTX 50 mg/m^2^ + IP PTX 20 mg/m^2^ (days 1, 8) + oral S-1 80 mg/m^2^ (days 1–14 of 21 days cycle)	OS: 16.3 months 1-year OS 62%, 2-year 23%; overall response rate 36%, disease control rate 82%	Encouraging outcomes; conversion surgery led to longer OS (27.8 vs. 14.2 months)
	Yamada et al. (2020) [4]	Phase I/II	IV GEM 800 mg/m^2^ + IV nab-PTX 75 mg/m^2^ + IP PTX 20 mg/m^2^ (days 1, 8, 15 of 21 days cycle)	RFS: 6 months OS: 14.5 months, response rate 50%, disease control rate 95%, cytology turned negative 39%	Good cytology conversion; OS longer in patients with conversion surgery
Gastric	Yang XJ et al. 2011 [78]	Phase III RCT	CRS alone vs. CRS + HIPEC (CDDP 120 mg + MMC 30 mg)	OS: 6.5 in CRS group vs. 11.0 months (CRS + HIPEC group) (*p* = 0.046)	First RCT showing HIPEC significantly improves OS in GC patients with PMs.
	Bonnot PE et al (2019) [23]	Phase III RCT	CRS alone vs. CRS + HIPEC (HIPEC regimen per study)	OS: 12.1 in CRS alone group vs. 18.8 months in CRS + HIPEC group, RFS: 7.8 vs. 13.6 months	Significantly better OS and RFS with HIPEC, especially in low-PCI and CC-0/1 patients
	Rau B et al (2024) [5]	Phase III RCT	CRS alone vs. CRS + HIPEC (CDDP 75 mg/m^2^ + MMC 15 mg/m^2^)	OS: 14.9 in CRS group vs 14.9 months in CRS + HIPEC group (*p* = 0.16); PFS: 3.5 vs. 7.1 months (*p* = 0.047)	PFS not OS significantly improved with HIPEC
	Ishigami H et al. (2010) [79]	Phase II	IV PTX 50 mg/m^2^ + IP PTX 20 mg/m^2^ (days 1, 8 of 21days cycle) + S-1 80 mg/m^2^ (days 1–14)	Median OS 22.5 months; 1-year OS 78%; overall response rate 56%; cytologyturned negative in 86%	High response rate and cytology clearance
	Ishigami H et al. (2018) [1]	Phase III RCT	IV PTX 50 mg/m^2^ + IP PTX 20 mg/m^2^ (days 1, 8 of 3 weeks cycle) + S-1 80 mg/m^2^ (days 1–21) in IP group vs. CDDP 60 mg/m^2^ (day 8 for a 5 weeks cycle) + S-1 80 mg/m^2^ (days 1 to 21) in SP group	OS: 15.2 in SP group vs. 17.7 months in IP group (*p* = 0.08) In sensitivity analysis adjusted for baseline ascites, OS in IP group is longer than SP group adjusted HR 0.59, *p* = 0.008.	Trend towards longer OS with IP group; benefit more apparent in adjusted analysis for ascites
Colorectal	Verwaal VJ et al. (2003) [3]	Phase III RCT	5FU-LV with or without palliative surgery vs. CRS + HIPEC with MMC +5-FU/LV	OS: 12.6 in 5FU-LV alone group vs. 22.3 months in HIPEC group (*p* = 0.032)	Significant survival benefit with CRS + HIPEC, especially in patients with limited tumor
	Quénet F et al. (2021) [94]	Phase III RCT	CRS alone vs. CRS + HIPEC with L-OHP	OS: 41.2 in CRS alone vs. 41.7 months in CRS + HIPEC group (*p* = n.s)	No additional OS benefit from CRS +HIPEC
Pseudomyxoma	Prabhu A et al. (2020) [102]	Phase II	Laparoscopic HIPEC with L-OHP 200 mg/m^2^ + IP docetaxel 40 mg/m^2^ + IP CDDP 40 mg/m^2^ + S-1	81.5% of patients qualified for CRS and HIPEC	High downstaging rate showed.

**Table 3:** Adverse Events by Cancer Type. n.s: not significant.

Tumor Type	Author, Year	Grade ≥ 3 Adverse Events (Type, %)	Catheter-Related Complications (%)	Treatment-Related Mortality
Ovarian	Alberts et al. (1996) [57]	Anemia 25% (IV group), vs. 26% (IP group), neutropenia 69 vs. 56%, leukopenia 50 vs. 40%, thrombocytopenia, 9 vs. 8%	5 patients (1.8%): Details are not reported.	0% in IV group vs. 0.7% due to respiratory failure and bronchopneumonia in IP group IV group
	Markman et al. (1998) [58]	Higher incidence of neutropenia, thrombocytopenia, and gastrointestinal and metabolic toxicities in IP group. (grade and % are not described)	— (not reported)	—
	Armstrong et al. (2006) [59]	Markedly increased incidences of fatigue (18%), pain (11%), infection (16%), fever (9%), leukopenia (76%), thrombocytopenia (12%), genitourinary event (7%), gastrointestinal event (46%), metabolic event (27%), pain (11%) and neurotoxicity (19%) in IP arm	Infection (10.2%), catheter obstruction (4.8%), catheter leak (1.4%), access problem (2.4%), fluid leak out vagina (0.4%)	1.9% in IV group vs. 2.4% in IP group. All cases are attributed to infection.
	Nagao et al. (2020) [2]	96% in IV group and 93.2% in IP group. Main AEs are anemia 67% vs. 64%, neutropenia 82% vs. 80%, thrombocytopenia 26% vs. 27%, abdominal pain 0% vs. 1.4%, nausea 2.7% vs. 1%, fatigue 1.3% vs. 1.7% and neuro system disorders 5.4% vs. 2.4%	catheter obstruction (2.7%), IP site leakage (5.7%)	0%
Pancreatic	Satoi et al. (2017) [70]	Hematologic AEs: neutropenia (42%), leukopenia (18%), febrile neutropenia (6%), and anemia (3%). Non-hematologic AEs: appetite loss (12%), nausea (9%), vomiting, diarrhea, mucositis (6%).	Infection (3%), dislocation of the device (6%)	3% due to superior mesenteric arterial thrombosis
	Yamada et al. (2020) [4]	Hematologic AEs: leucocytopenia (48%), neutropenia (70%), febrile neutropenia (9%), anaemia (17%) and thrombocytopenia (13%). Non-hematologic AEs: appetite loss (9%) and nausea (4%).	1 patient (2.1%): Details are not reported.	—
Gastric	Yang XJ et al. 2011 [78]	4 patients (11.7%) in CRS group vs. 5 (14.7%) patients in the CRS + HIPEC group (*p* = n.s). The contents are infection, respiratory failure, gastrointestinal bleeding, bone marrow suppression and intestinal obstruction.	—	—
	Bonnot PE et al (2019) [23]	55.3% in CRS alone vs. 53.7% in CRS + HIPEC (*p* = 0.49): Details are not reported.	—	90-days mortality: 7.4% in CRS alone vs. 10.1% in CRS + HIPEC (*p* = 0.82)
	Rau B et al (2024) [5]	Similar incidence: 38.1% in CRS alone vs. 43.6% in CRS + HIPEC (*p* = 0.79).	—	0% in CRS alone vs. 4% in CRS + HIPEP (*p* = 0.49)
	Ishigami H et al. (2010) [79]	Neutropenia 38%, leukopenia 18% and anemia 10%	1 patient (2.5%): catheter obstruction	0%
	Ishigami H et al. (2018) [1]	Leukopenia 9% in SP group vs. 25% in IP group (*p* = 0.02), neutropenia 30% vs. 50% (*p* = 0.02); nonhematologic AEs are no differences between two group.	port infection 2.5%, catheter obstruction 2.5%, subcutaneous hematoma 0.8% and fistula between the catheter and small intestine 0.8%	0%
Colorectal	Verwaal VJ et al. (2003) [3]	Fever 6%, leukopenia 17%, thrombocytopenia 4%, neuropathy 4%, pulmonary embolus 4%, renal obstruction 4%, heart failure 12%, gastrointestinal fistula 15%, hemorrhage 14%, psychological disorders 10% in HIPEC group	Catheter infections 6%	8% due to abdominal pain followed by HIPEC (2%) or postoperative complications (2%)
	Quénet F et al. (2021) [94]	The incidences at 30 days were no difference between 32% in CRS alone group and 42% in CRS + HIPEC group (*p* = 0.08). However, at 60 days, events in CRS + HIPEC group were more than CRS alone (26% vs. 15%, *p* = 0.03)	—	At 30 days, 2 patients (1.5%) in each group. At 60 days, one additional patients (total 2.2%) in CRS alone group due to acute respiratory distress and two patients (total 3%) in CRS alone group due to pulmonary embolism and bilateral pneumonia.
Pseudomyxoma	Prabhu A et al. (2020) [102]	Major morbidity 13.6%	6% details are not reported.	4.5% details are not reported.

## Data Availability

No new data were created or analyzed in this study. Data sharing is not applicable to this article.

## Abbreviations

The following abbreviations are used in this manuscript:

PMs	Peritoneal metastases
IPC	Intraperitoneal chemotherapy
IP	Intraperitoneal
IV	Intravenous
iPocc	Intraperitoneal Therapy for Ovarian Cancer with Carboplatin
HIPEC	Hyperthermic intraperitoneal chemotherapy
PIPAC	Pressurized intraperitoneal aerosol chemotherapy
RCTs	Randomized controlled trials
CRS	Cytoreductive surgery
GC	Gastric cancer
OS	Overall survival
CTCAE	Common Terminology Criteria for Adverse Events
TME	Tumor microenvironment
PDS	Primary debulking surgery
CDDP	Cisplatin
CPA	Cyclophosphamide
PTX	Paclitaxel
CBDCA	Carboplatin
AUC	Area under curve
PFS	Progression free survival
MA	Malignant ascites
FOLFORINOX	Fluorouracil, leucovorin, irinotecan, and oxaliplatin
GEM	Gemcitabine
FN	Febrile neutropenia
MMC	Mitomycin C
NRCT	Nonrandomized control trials
RR	Risk ratio
CRC	Colorectal cancer
Bev	Bevacizumab
5-FU	5-fluorouracil
OR	Odds ratio
PMP	Pseudomyxoma peritonei
EPIC	Early postoperative intraperitoneal chemotherapy
ECM	Extracellular matrix
L-OHP	Oxaliplatin
Doxo	Doxorubicin
CPT-11	Irinotecan
HCC	Hepatocellular carcinoma
